# Supplementary material for: Identification and characterization of a SARS-CoV-2 specific CD8+ T cell response with immunodominant features
Source: Nat Commun. 2021 May 10;12:2593. doi: 10.1038/s41467-021-22811-y (PMC8110804; doi:10.1038/s41467-021-22811-y)
Supplement: Supplementary file 1 — Supplementary Information [file 41467_2021_22811_MOESM1_ESM.pdf]

Supplementary Information

Identification and characterization of a SARS-CoV-2 specific CD8 T cell response with immunodominant features

Anastasia Gangaev[1], Steven L. C. Ketelaars[1], Olga I. Isaeva[1], Sanne Patiwael[1], Anna Dopler[1], Kelly Hoefakker[1], Sara De Biasi[2], Lara Gibellini[2], Cristina Mussini[2], Giovanni Guaraldi[2], Massimo Girardis[2], Cami M. P. Talavera Ormeno[3], Paul J. M. Hekking[3], Neubury M. Lardy[4], Mireille Toebes[1], Robert Balderas[5], Ton N. Schumacher[1], Huib Ovaa[3], Andrea Cossarizza[2], Pia Kvistborg[1*]

[1] Division of Molecular Oncology and Immunology, The Netherlands Cancer Institute, Amsterdam, North Holland, 1066 CX, The Netherlands

[2] Department of Medical and Surgical Sciences for Children and Adults, University of Modena and Reggio Emilia School of Medicine, Modena, Emilia Romagna, 41125, Italy

[3] Department of Cell and Chemical biology, Leiden University Medical Center, Leiden, South Holland, 2300 RC, The Netherlands

[4] Department of Immunogenetics, Sanquin Diagnostics B.V., Amsterdam, North Holland, 1066 CX, The Netherlands

[5] Department of Biological Sciences, BD Bioscience, San Jose, CA 95131, USA.

[*]Address correspondence to Pia Kvistborg (p.kvistborg@nki.nl)

These authors contributed equally to this work: Anastasia Gangaev, Steven L. C. Ketelaars
These authors jointly supervised this work: Andrea Cossarizza, Pia Kvistborg

Supplementary Figures

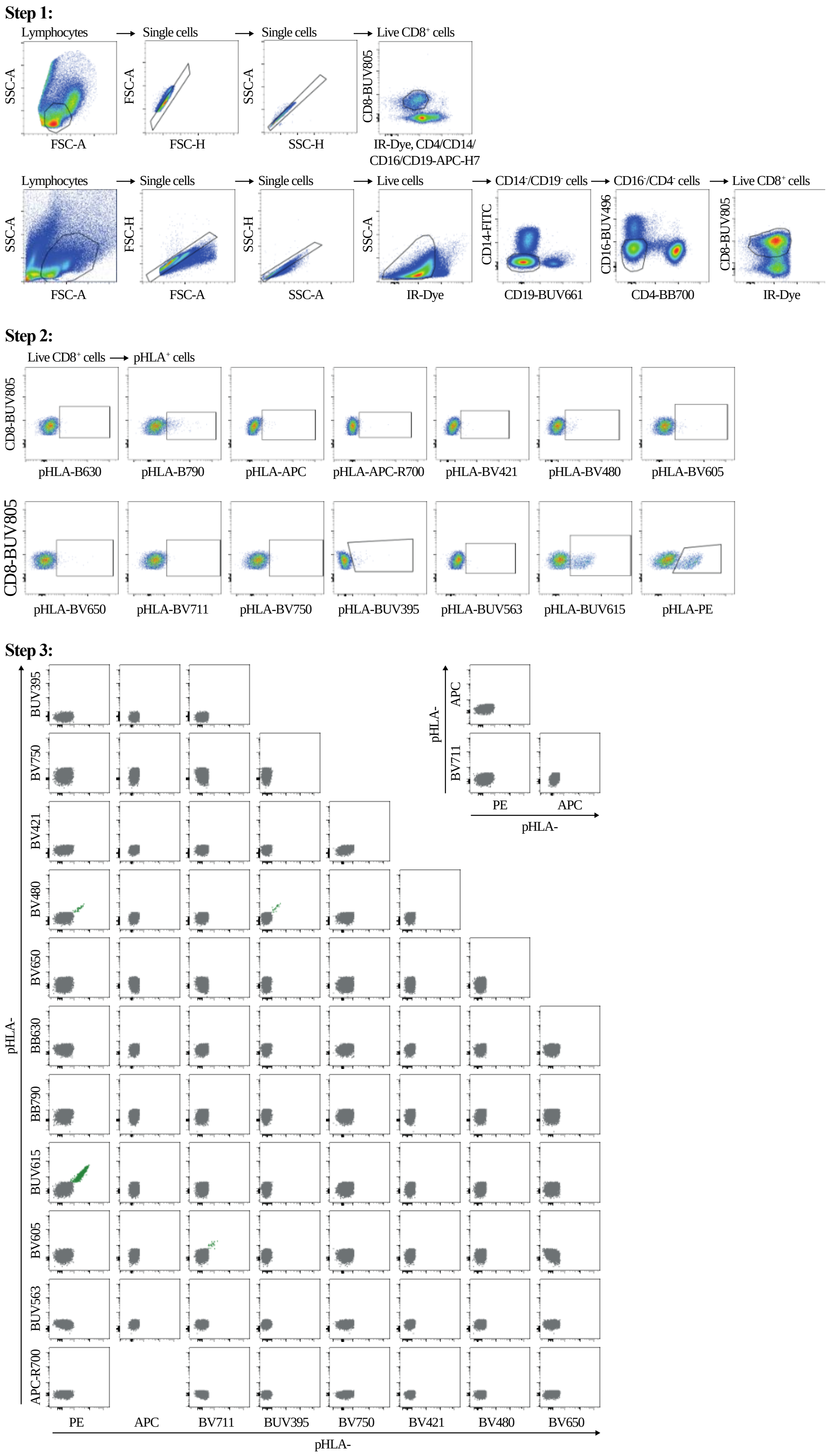

Supplementary Fig. 1: Representative gating strategy to identify SARS-CoV-2-specific CD8 T cell responses presented in Fig. 1c-e and Fig. 5a and b. Step 1: Gating strategy used to identify live $CD8^+$ cells. Step 2: Gating strategy used to identify $pHLA^+$ cells within the live $CD8^+$ cell population. Step 3: Representative overview of all 75 pHLA dual color code combinations. Boolean gating. Was used to identify SARS-CoV-2-specific CD8 T cells (double-positive $pHLA^+$ $CD8^+$ cells, green) and bulk CD8 T cells ($pHLA^-$ $CD8^+$ cells, grey). pHLA: peptide-human leukocyte antigen.

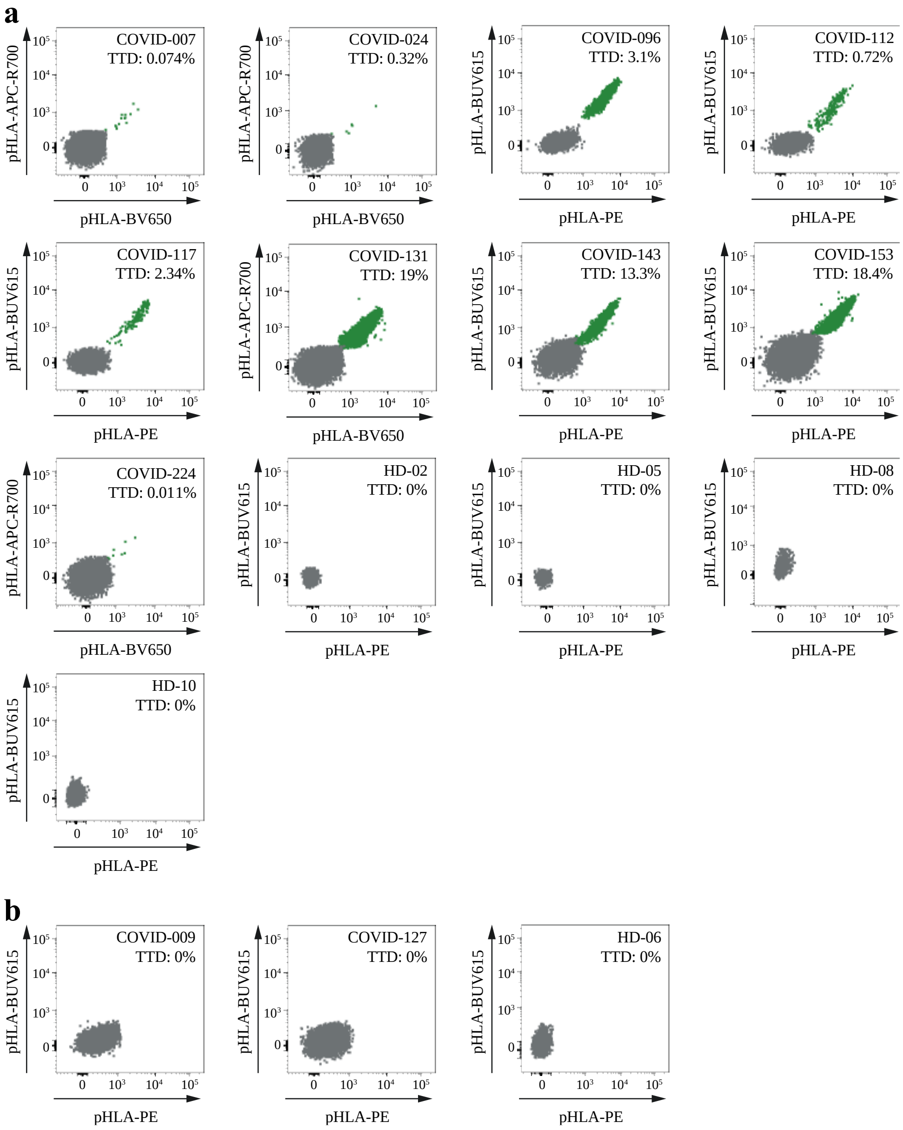

Supplementary Fig. 2: Flow cytometry plots of the TTD-specific CD8 T cell responses presented in Fig. 2a-c. **a** Dot plots of TTD-specific CD8 T cell responses detected in HLA-A*01:01-positive donors. Magnitude of TTD-specific CD8 T cell responses is represented as the percentage of double-positive $pHLA^+$ cells (green) of total $CD8^+$ cells (grey). Representative gating strategy is provided in Supplementary Fig. 1. **b** Dot plots representing the negative control for the detected TTD-specific CD8 T cell responses in HLA-A*01:01-negative donors. The magnitude of the TTD-specific CD8 T cell responses is represented as the percentage of double-positive $pHLA^+$ cells (green) of total $CD8^+$ cells (grey). Representative gating strategy is provided in Supplementary Fig. 1. pHLA: peptide-human leukocyte antigen, TTD: TTDPSFLGRY, HD: healthy donor.

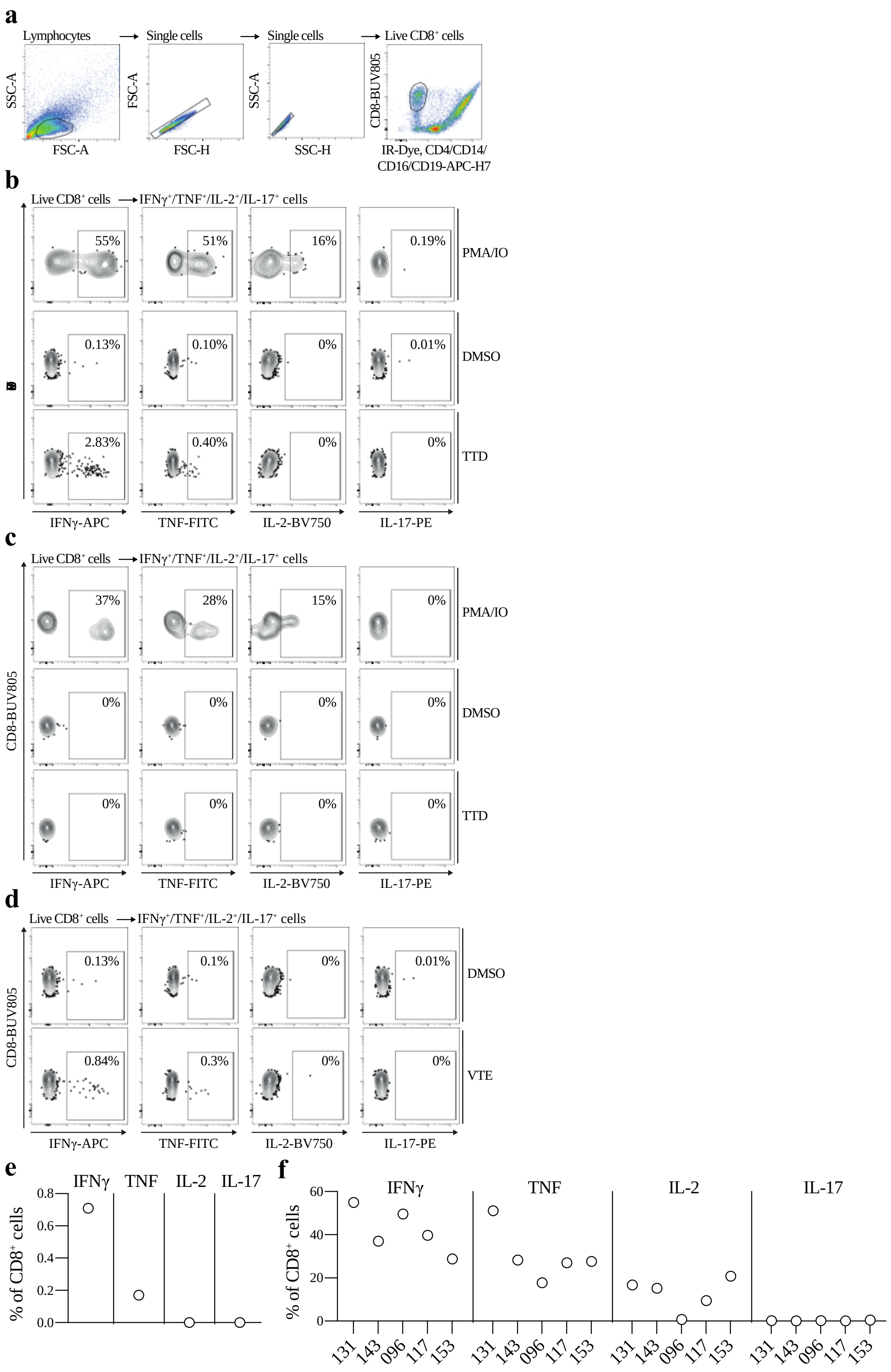

Supplementary Fig. 3: Representative gating strategy used for the functional assessment of SARS-CoV-2-specific CD8 T cells presented in Fig. 3a and b and Fig. 5g and h. **a** Gating strategy used to identify live CD8$^+$ cells. **b** Representative gating strategy used to assess the expression of IFNγ, TNF, IL-2 and IL-17 in CD8 T cells from patient COVID-131 after

stimulation with PMA/IO (technical control, top row), DMSO (negative control, middle row) and the SARS-CoV-2 epitope TTD (bottom row). Percentages represent the frequency of cytokine producing cells. The gates were set relative to the DMSO and adjusted for the PMA/IO control if needed. **c** Representative flow cytometry plots illustrating the gating strategy used to assess the expression of IFNγ, TNF, IL-2 and IL-17 production in CD8 T cells from COVID-143 after stimulation with PMA/IO (technical control, top row), DMSO (negative control, middle row) and the SARS-CoV-2 epitope TTD (bottom row). Percentages represent the frequency of cytokine producing cells. The gates are based on the DMSO control and adjusted for the PMA/IO control if needed. **d** Representative gating strategy used to assess the expression of IFNγ, TNF, IL-2 and IL-17 in CD8 T cells from patient COVID-131 after stimulation with VTE (HLA-A*01:01-restricted CMV epitope, bottom row), DMSO (negative control, top row). Percentages represent the frequency of cytokine producing cells. The gates were set relative to the DMSO and adjusted for the PMA/IO control if needed. **e** Expression of IFNγ, TNF, IL-2 and IL-17 in CD8 T cells from COVID-131 with acute disease after stimulation with VTEHDTLLY (HLA-A*01:01-restricted CMV epitope) for 12h. Percentages represent the frequency of cytokine producing cells after subtracting the percentages of the DMSO control. **f** Expression of IFNγ, TNF, IL-2 and IL-17 in CD8 T cells from COVID-19 patients (n=5) with acute disease after stimulation with PMA/IO (technical control) for 12h. Percentages represent the frequency of cytokine producing cells after subtracting the percentages of the DMSO control. TTD: TTDPSFLGRY, VTE: VTEHDTLLY.

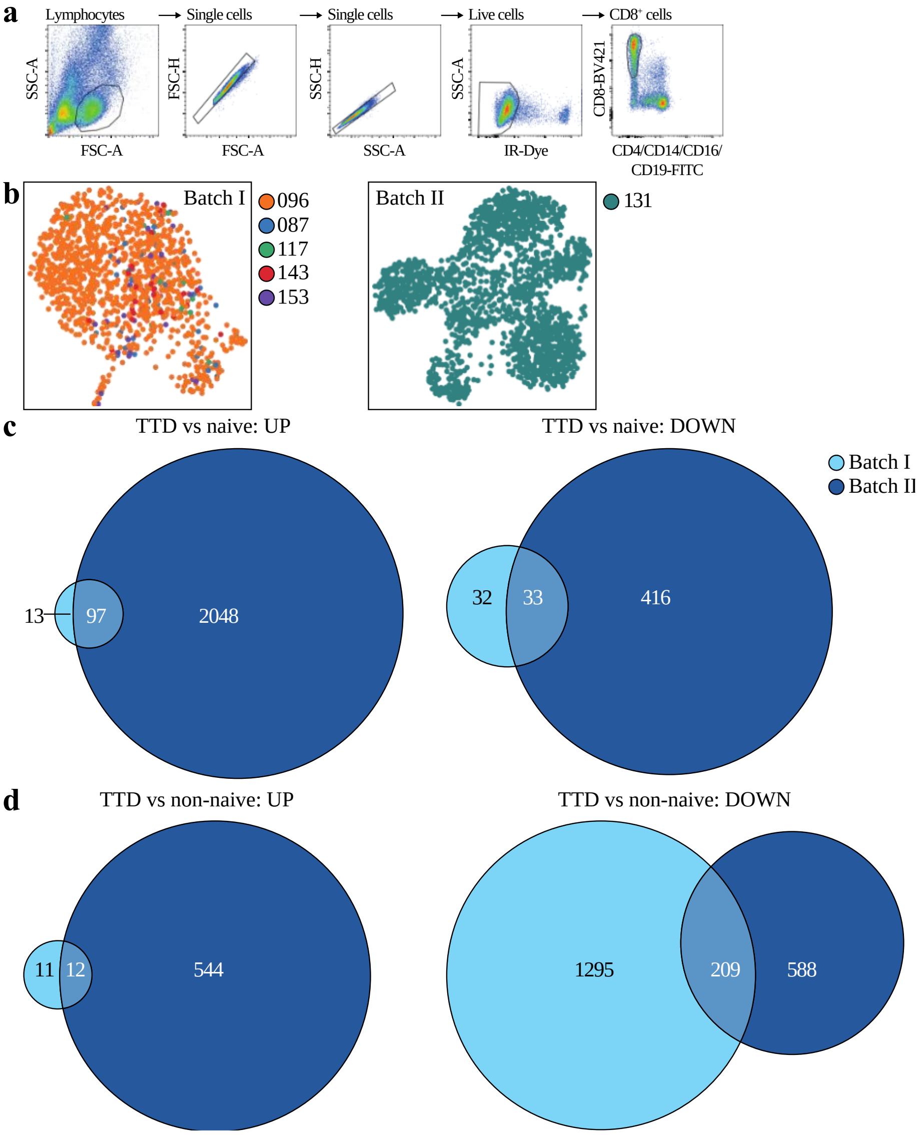

Supplementary Fig. 4: Gene expression analysis of CD8 T cells from COVID-19 patients presented in Fig. 4. **a** Representative gating strategy used to sort on live CD8^{+} cells for single-cell RNA and TCR sequencing. **b** UMAP representations of single-cell gene expression data of CD8 T cells (batch I: n=1180 and batch II: n=1884) isolated from COVID-19 patients (batch I: n=5 and batch II: n=1). Detailed information about the number of patients and cells for each individual patient/batch is provided in Table 2. **c** Number of differentially expressed genes in TTD-specific CD8 T cells compared to bulk naïve CD8 T cells. Proportional Venn diagrams illustrate the overlap and differences in the number of genes that were found to be differentially expressed in batch I (light blue) compared to batch II (dark blue). **d** Number of differentially expressed genes in TTD-specific CD8 T cells compared to bulk non-naïve CD8 T cells. Proportional Venn diagram illustrate the overlap and differences in the number of genes that were found to be differentially expressed batch I (light blue) compared to batch II (dark blue).

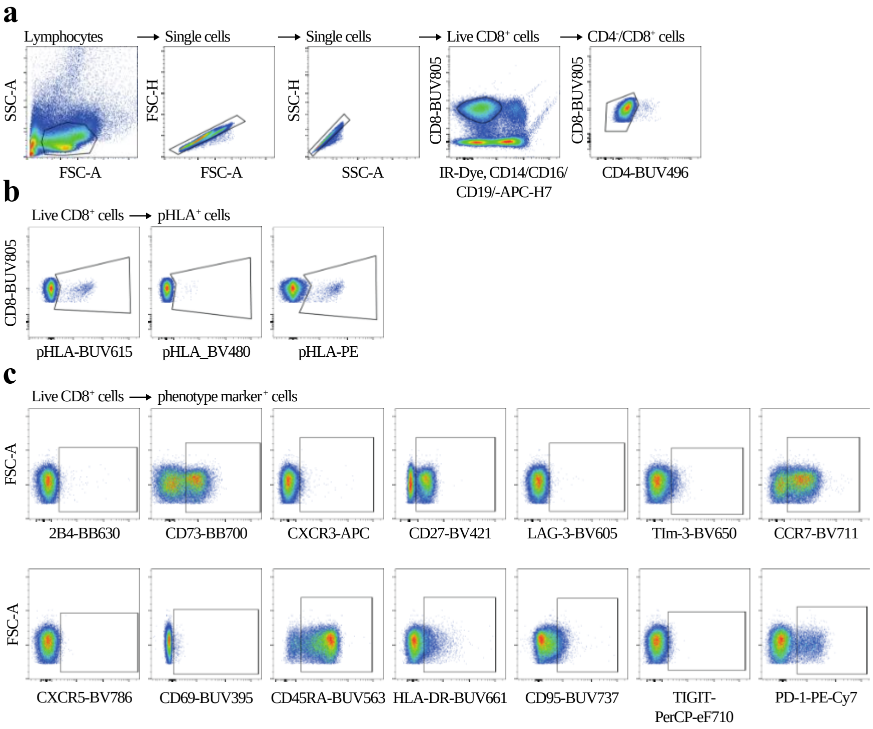

Supplementary Fig. 5: Representative gating strategy used for phenotypic characterization presented in Fig. 5c-f. **a** Gating strategy used to identify live CD8$^+$ cells. **b** Gating of pHLA$^+$ cells within the live CD8$^+$ cell population to identify SARS-CoV-2-specific CD8 T cells as shown in Supplementary Fig. 1. **c** Representative gating on live CD8$^+$ cells that were used to assess expression levels of phenotypic markers on SARS-CoV-2-specific CD8 T cells.

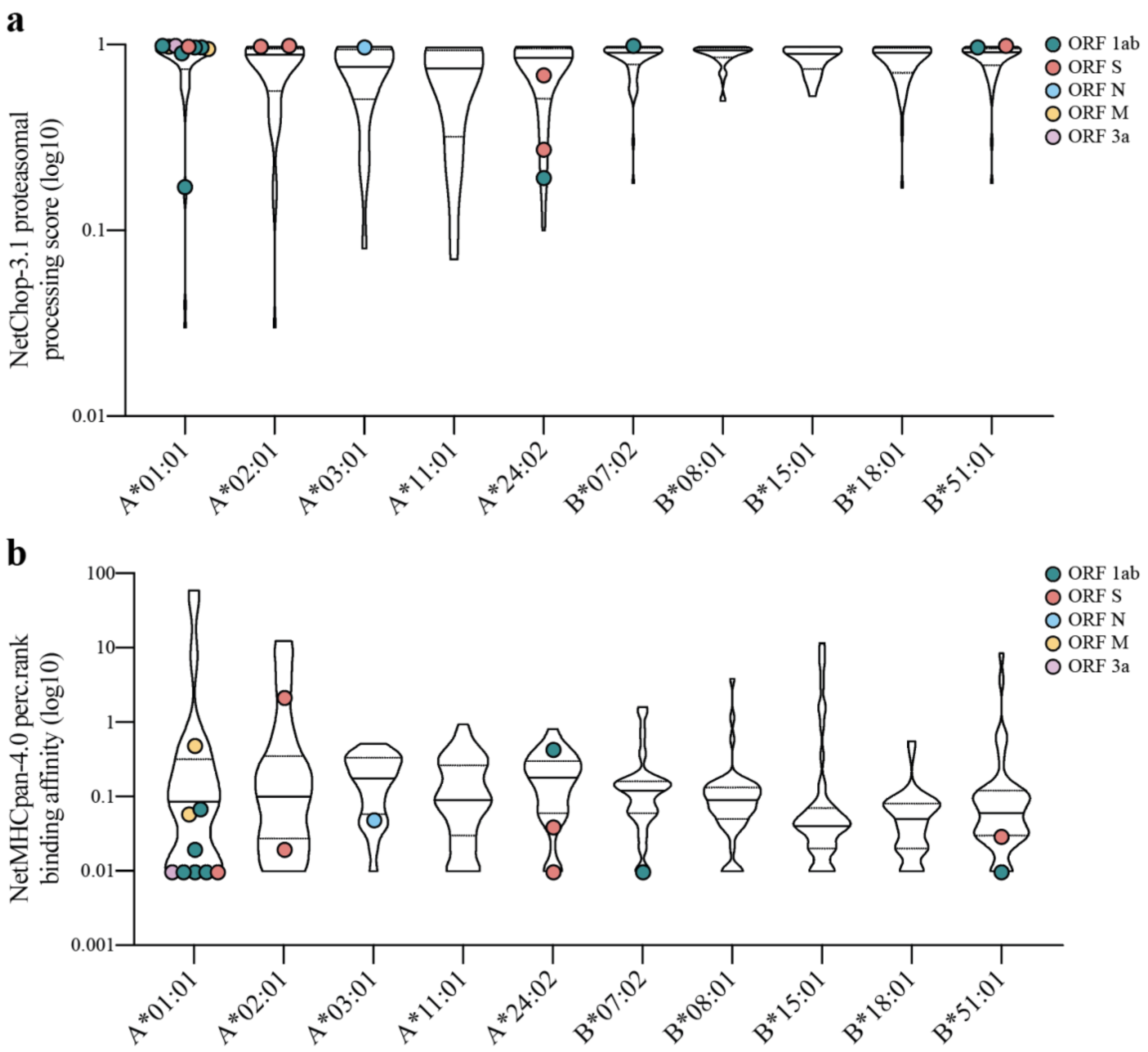

Supplementary Fig. 6: Prediction quality of selected SARS-CoV-2 epitopes. **a** Violin plots representing the distribution of the proteasomal processing scores predicted by Netchop-3.1 for the 50 selected SARS-CoV-2 epitopes per HLA allele included in this study. Colored dots represent CD8 T cell recognized epitopes (n=18) and provide information about their protein origin. **b** Violin plots representing the distribution of the percentile rank binding affinity as predicted by NetMHCpan-4.0 for the 50 selected SARS-CoV-2 epitopes per HLA allele included in this study. Colored dots represent CD8 T cell recognized epitopes (n=18) and provide information about their protein origin. ORF: open reading frame, S: spike, N: nucleoprotein, M: membrane.

Supplementary Tables

Supplementary Table 1: Characteristics of COVID-19 patients and healthy donors included in the study. Covered HLA alleles are indicated (*). Patients were excluded from the analysis of SARS-CoV-2 CD8 T cells if: (1) none of the HLA alleles were covered, (2) <1000 CD8 T cells were acquired, and (3) HLA typing failed.

Patient	Disease Status	Gender	Age range	Treatment	Hospitalization (weeks)	Outcome	Treatment prior to sampling (days)	Hospitalization prior to sampling (days)	HLA-A	HLA-A	HLA-B	HLA-B	Analysis of SARS-CoV-2 CD8 T cell responses
COVID-024	Critical	Male	70-80	Anakinra	4	Discharged	2	2	01:01*	30:02	18:01*	57:02	Yes
COVID-040	Critical	Male	60-70	N/A	6	Deceased	N/A	3	02:01*	66:01	18:01*	41:02	No (2)
COVID-042	Critical	Female	50-60	Anakinra	11	Transferred	2	-2	03:01*	24:02*	35:02	-	Yes
COVID-084	Critical	Male	60-70	Tocilizumab	3	Discharged	18	17	02:01*	03:01*	08:01*	35:02	Yes
COVID-094	Critical	Female	70-80	Tocilizumab	2	Deceased	13	17	02:01*	32:01	51:08	56:01	Yes
COVID-112	Critical	Male	70-80	Tocilizumab	5	Discharged	12	20	01:01*	26:01	35:02	35:03	Yes
COVID-121	Critical	Male	30-40	Tocilizumab	3	Discharged	8	9	03:01*	74:01	07:02*	42:01	Yes
COVID-123	Critical	Male	70-80	Tocilizumab	6	Deceased	13	14	03:01*	32:01	27:05	51:01*	Yes
COVID-127	Critical	Male	70-80	Tocilizumab	3	Deceased	2	2	29:02	31:01	35:01	44:03	No (1)
COVID-129	Critical	Male	70-80	Tocilizumab	20	Transferred	15	18	02:01*	29:02	15:01*	44:03	Yes
COVID-131	Critical	Male	40-50	Tocilizumab	3	Discharged	7	10	01:01*	68:01	15:17	41:01	Yes
COVID-140	Critical	Male	50-60	Tocilizumab	5	Deceased	6	24	11:01*	68:02	35:01	53:01	Yes
COVID-141	Critical	Male	70-80	Tocilizumab	28	Discharged	6	11	11:01*	68:01	35:03	50:01	Yes
COVID-143	Critical	Male	70-80	Tocilizumab	2	Discharged	10	12	01:01*	02:01*	44:03	51:01*	Yes
COVID-147	Critical	Male	70-80	Tocilizumab	18	Discharged	6	10	02:01*	26:01	38:01	44:02	Yes
COVID-150	Critical	Male	70-80	Anakinra	13	Deceased	14	14	24:02*	29:01	13:02	35:03	Yes
COVID-152	Critical	Male	60-70	Tocilizumab	2	Deceased	1	22	01:01*	30:01	13:02	53:01	No (2)
COVID-180	Critical	Male	40-50	Tocilizumab	4	Deceased	5	8	24:07	24:07	35:05	35:05	No (1)
COVID-007	Severe	Female	30-40	Tocilizumab	4	Deceased	1	5	01:01*	32:01	39:01	57:01	Yes
COVID-009	Severe	Male	60-70	Tocilizumab	3	Discharged	3	6	02:05	32:01	13:02	14:02	No (1)
COVID-015	Severe	Male	50-60	Tocilizumab	14	Discharged	2	1	02:01*	24:02*	18:01*	35:03	Yes
COVID-033	Severe	Female	70-80	Tocilizumab	3	Discharged	0	1	02:01*	68:01	35:01	49:01	Yes
COVID-087	Severe	Female	50-60	Tocilizumab	12	Discharged	8	8	02:01*	03:02	39:01	50:01	Yes
COVID-096	Severe	Male	40-50	Tocilizumab	4	Discharged	7	7	01:01*	23:01	49:01	57:01	Yes
COVID-111	Severe	Male	60-70	Tocilizumab	9	Discharged	7	9	02:01*	30:02	18:01*	51:01*	Yes
COVID-116	Severe	Male	80-90	Tocilizumab	4	Discharged	1	5	01:01*	26:01	13:02	44:02	No (2)
COVID-117	Severe	Male	60-70	Tocilizumab	2	Discharged	1	2	01:01*	31:01	15:17	38:01	Yes
COVID-153	Severe	Male	50-60	Tocilizumab	3	Discharged	7	9	01:01*	02:01*	35:01	35:02	Yes
COVID-166	Severe	Male	70-80	Tocilizumab	1	Discharged	15	29	02:01*	32:01	27:05	35:02	Yes
COVID-174	Severe	Male	80-90	Tocilizumab	16	Discharged	8	15	02:01*	24:02*	08:01*	44:02	Yes
COVID-002	Moderate	Male	80-90	None	3	Deceased	0	2	03:02	32:01	18:01*	44:02	Yes
COVID-004	Moderate	Male	30-40	None	1	Discharged	0	3	03:01*	24:02*	07:02*	35:01	Yes
COVID-218	Asymptomatic	Male	30-40	None	N/A	N/A	N/A	N/A	03:01*	26:01	18:01*	38:01	Yes
COVID-219	Asymptomatic	Male	20-30	None	N/A	N/A	N/A	N/A	02:01*	03:01*	18:01*	41:01	Yes
COVID-220	Asymptomatic	Male	20-30	None	N/A	N/A	N/A	N/A	-	-	-	-	No (3)
COVID-221	Asymptomatic	Male	30-40	None	N/A	N/A	N/A	N/A	30:01	74:01	18:01*	51:01*	Yes
COVID-222	Asymptomatic	Male	30-40	None	N/A	N/A	N/A	N/A	24:02*	26:01	07:02*	38:01	Yes
COVID-223	Asymptomatic	Male	50-60	None	N/A	N/A	N/A	N/A	32:01	32:01	15:17	49:01	No (1)
COVID-224	Asymptomatic	Male	30-40	None	N/A	N/A	N/A	N/A	01:01*	30:01	08:01*	39:01	Yes
HD-01	Healthy	Male	70-80	None	N/A	N/A	N/A	N/A	02:01*	03:01*	15:01*	35:01	Yes
HD-02	Healthy	Male	70-80	None	N/A	N/A	N/A	N/A	01:01*	03:01*	07:02*	40:01	Yes
HD-03	Healthy	Female	70-80	None	N/A	N/A	N/A	N/A	03:01*	-	35:01	-	Yes
HD-05	Healthy	Male	50-60	None	N/A	N/A	N/A	N/A	01:01*	11:01*	08:01*	38:01	Yes
HD-06	Healthy	Male	60-70	None	N/A	N/A	N/A	N/A	02:05	11:01*	40:01	50:01	Yes
HD-08	Healthy	Female	60-70	None	N/A	N/A	N/A	N/A	01:01*	23:01	-	41:02	Yes
HD-10	Healthy	Female	60-70	None	N/A	N/A	N/A	N/A	01:01*	-	35:01	44:03	Yes

Supplementary Table 2: Detected SARS-CoV-2-specific CD8 T cell responses. Magnitude represents the percentage of $pHLA^{+}$ cells of total CD8 T cells. ORF: open reading frame, S: spike, N: nucleoprotein, M: membrane.

Patient	Disease status	Epitope	Epitope origin	HLA restriction	Magnitude (% of pHLA+ cells of total CD8+ cells)
COVID-024	Critical	TTDPSFLGRY	ORF 1ab	A*01:01	0.320
COVID-042	Critical	QYIKWPWYI	ORF S	A*24:02	0.006
COVID-094	Critical	YLQPRTFLL	ORF S	A*02:01	0.085
COVID-112	Critical	TTDPSFLGRY	ORF 1ab	A*01:01	0.720
COVID-131	Critical	ATSRTLSYY	ORF M	A*01:01	0.021
COVID-131	Critical	CTDDNALAYY	ORF 1ab	A*01:01	0.017
COVID-131	Critical	FTSDYYQLY	ORF 3a	A*01:01	0.377
COVID-131	Critical	LTDEMIAQY	ORF S	A*01:01	0.020
COVID-131	Critical	PTDNYITTY	ORF 1ab	A*01:01	0.390
COVID-131	Critical	TTDPSFLGRY	ORF 1ab	A*01:01	19.000
COVID-143	Critical	CTDDNALAYY	ORF 1ab	A*01:01	0.210
COVID-143	Critical	DTDFVNEFY	ORF 1ab	A*01:01	0.270
COVID-143	Critical	PTDNYITTY	ORF 1ab	A*01:01	0.580
COVID-143	Critical	TTDPSFLGRY	ORF 1ab	A*01:01	13.300
COVID-007	Severe	FTSDYYQLY	ORF 3a	A*01:01	0.062
COVID-007	Severe	TTDPSFLGRY	ORF 1ab	A*01:01	0.074
COVID-087	Severe	RLNEVAKNL	ORF S	A*02:01	0.017
COVID-087	Severe	YLQPRTFLL	ORF S	A*02:01	0.038
COVID-096	Severe	PTDNYITTY	ORF 1ab	A*01:01	0.057
COVID-096	Severe	TTDPSFLGRY	ORF 1ab	A*01:01	3.100
COVID-096	Severe	TVATSRTLSY	ORF M	A*01:01	0.100
COVID-111	Severe	LPYPDPSRI	ORF 1ab	B*51:01	0.220
COVID-111	Severe	YLQPRTFLL	ORF S	A*02:01	0.210
COVID-117	Severe	TTDPSFLGRY	ORF 1ab	A*01:01	2.340
COVID-153	Severe	PTDNYITTY	ORF 1ab	A*01:01	0.085
COVID-153	Severe	TTDPSFLGRY	ORF 1ab	A*01:01	18.400
COVID-004	Moderate	KTFPPTEPK	ORF N	A*03:01	0.038
COVID-219	Asymptomatic	KTFPPTEPK	ORF N	A*03:01	0.125
COVID-221	Asymptomatic	IPTNFTISV	ORF S	B*51:01	0.018
COVID-222	Asymptomatic	IPRRNVATL	ORF 1ab	B*07:02	0.009
COVID-222	Asymptomatic	NYNYLYRLF	ORF S	A*24:02	0.009
COVID-222	Asymptomatic	QYIKWPWYI	ORF S	A*24:02	0.011
COVID-222	Asymptomatic	VQSTQWSLF	ORF 1ab	A*24:02	0.005
COVID-224	Asymptomatic	LTGHMLDMY	ORF 1ab	A*01:01	0.010
COVID-224	Asymptomatic	TTDPSFLGRY	ORF 1ab	A*01:01	0.011
HD-01	Healthy	ILMTARTVY	ORF 1ab	B*15:01	0.008

Supplementary Table 3. Characteristics of CD8 T cell recognized SARS-CoV-2 epitopes. Epitopes with reported hotspot mutations (SNV frequency cutoff: 2.5%) are indicated (*). AA: amino acid, NT: nucleotide, ORF: open reading frame, S: spike, N: nucleoprotein, M: membrane.

Epitope	AA length	Peptide origin	HLA restriction	Patients with detectable CD8 T cell responses (n, %)	SARS-CoV-1	HCoV-NL63	HCoV-229E	HCoV-OC43	HCoV-HKU1	AA location	NT location	Mutation	Alternative epitope	SNV frequency
ATSRTLSYY	9	ORF M	A*01:01	1/9 (11%)	Yes	No	No	No	No	171-179	27033-27059	T175M	ATSRMLSYY	0.332
CTDDNALAYY	10	ORF1ab	A*01:01	2/9 (22%)	Yes	No	No	No	No	4163-4172	12752-12781	T4164I	CIDDNALAYY	0.051
												T4164A	CADDNALAYY	0.017
												D4165G	CTGDNALAYY	0.052
DTDFVNEFY	9	ORF1ab	A*01:01	1/9 (11%)	No	No	No	No	No	5130-5138	15652-15678	D5130Y	YTDFVNEFY	0.267
												T5131I	DIDFVNEFY	0.398
												E5136D	DTDFVNDFY	0.103
FTSDYYQLY	9	ORF3a	A*01:01	2/9 (22%)	No	No	No	No	No	207-215	26011-26037	F207L	LTSDYYQLY	0.032
												D210Y	FTSYYYQLY	0.022
												Q213K	FTSDYYKLY	0.080
												Q213H	FTSDYYHLY	0.010
IPRRNVATL	9	ORF1ab	B*07:02	1/3 (33%)	Yes	No	No	No	No	5916-5924	18010-18036	A5922S	IPRRNVSTL	1.730
												A5922V	IPRRNVVTL	0.100
												T5923I	IPRRNVAIL	0.182
IPTNFTISV	9	ORF S	B*51:01	1/4 (25%)	No	No	No	No	No	714-722	23702-23728	T716I	IPINFTISV	8.439*
												T719I	IPTNFIISV	0.022
KTFPPTEPK	9	ORF N	A*03:01	2/7 (28%)	Yes	No	No	No	No	361-369	29354-29380	T362K	KKFPPTEPK	0.015
												T362I	KIFPPTEPK	0.114
												P364S	KTFSPTEPK	0.031
												P364L	KTFLPTEPK	0.063
												P365L	KTFPLTEPK	0.062
												P365S	KTFPSTEPK	2.051
												T366R	KTFPPREPK	0.014
												T366I	KTFPPIEPK	0.087
												E367D	KTFPPTDPK	0.022
												P368L	KTFPPTELK	0.012
												P368S	KTFPPTESK	0.028
LPYPDPSRI	9	ORF1ab	B*51:01	1/4 (25%)	Yes	Yes	Yes	No	Yes	5221-5229	15925-15951	L5221F	FPYPDPSRI	0.041
												P5222S	LSYPDPSRI	0.021
LTDEMIAQY	9	ORF S	A*01:01	1/9 (11%)	No	No	No	No	No	865-873	24155-24181	I870V	LTDEMVAQY	0.029
												A871V	LTDEMIVQY	0.014
												A871S	LTDEMISQY	0.026
LTGHMLDMY	9	ORF1ab	A*01:01	1/9 (11%)	Yes	No	No	No	No	5287-5295	16123-16149	-	-	-
NYNYLYRLF	9	ORF S	A*24:02	1/6 (16%)	No	No	No	No	No	448-456	22904-22930	L452M	NYNYMYRLF	0.024
												L452R	NYNYRYRLF	0.327
												Y453F	NYNYLFRLF	0.342
												L455F	NYNYLYRFF	0.014
PTDNYITTY	9	ORF	A*01:01	4/9 (44%)	No	No	No	No	No	1321-1329	4226-4252	P1321S	STDNYITTY	0.170
												T1322I	PIDNYITTY	0.013
												T1322P	PPDNYITTY	0.010
												D1323Y	PTYNYITTY	0.040
												N1324S	PTDSYITTY	0.014
QYIKWPWYI	9	ORF S	A*24:02	2/6 (33%)	No	No	No	No	No	1208-1216	25184-25210	Q1208H	HYIKWPWYI	0.037
RLNEVAKNL	9	ORF S	A*02:01	1/13 (7%)	Yes	No	No	No	No	1185-1193	25115-25141	N1187Y	RLYEVAKNL	0.036
												K1191N	RLNEVANNL	0.095
TTDPSFLGRY	10	ORF1ab	A*01:01	9/9 (100%)	No	No	No	No	No	1637-1646	5174-5203	T1637I	ITDPSFLGRY	0.140
												T1638I	TIDPSFLGRY	0.020
												P1640L	TTDLSFLGRY	0.105
												P1640S	TTDSSFLGRY	0.229
												P1640H	TTDHSFLGRY	0.090
TVATSRTLSY	10	ORF M	A*01:01	1/9 (11%)	Yes	No	No	No	No	169-178	27027-27056	T175M	TVATSRMLSY	0.332
VQSTQWSLF	9	ORF1ab	A*24:02	1/6 (16%)	Yes	No	No	No	No	3595-3603	11048-11074	V3595G	GQSTQWSLF	0.010
												L3602F	VQSTQWSFF	0.016
YLQPRTFLL	9	ORF S	A*02:01	3/13 (23%)	No	No	No	No	No	269-277	22367-22393	P272L	YLQLRTFLL	0.820

Supplementary Table 4: Overview of UV-sensitive peptides used for generating the corresponding UV-sensitive pHLA monomers. ‘J’ indicates the UV-sensitive amino acid.

HLA allele	Conditional ligand
A*01:01	STAPG-J-LEY[1]
A*02:01	KILGFVF-J-V[2]
A*03:01	RIYR-J-GATR[1]
A*11:01	AIFQSS-J-TK[1]
A*24:01	VYG-J-VRACL[3]
B*07:02	AARG-J-TLAM[1]
B*08:01	FLRGRA-J-GL[2]
B*15:01	ILGP-J-GSVY[2]
B*18:01	SELE-J-KRY
B*51:01	IPT-J-FSISI

Supplementary Table 5: Streptavidin reagents used to generate fluorescent pHLA multimers.

Florochrome / DNA oligo	Manufacturer	Cat. No.	Lot. No.	Dilution
APC	Invitrogen	S868	1876191	1/16
APC-R700	BD	565144	9023546	1/10
BB630	BD	custom	0091407	1/10
BB790	BD	custom	0091415	1/10
BUV395	BD	564176	9078721	1/5
BUV563	BD	567655	9227337	1/6
BUV615	BD	613013	9193969	1/10
BV421	BD	563259	9197684	1/5
BV480	BD	564876	9115527	1/5
BV605	BD	563260	9119807	1/5
BV650	BD	563855	9197600	1/5
BV711	BD	563262	0007729	1/8
BV750	BD	custom	0091421	1/10
PE	Invitrogen	S866	1736956	1/9
Total-Seq-C0971	Biolegend	405271	B295732	1/50
Total-Seq-C0972	Biolegend	405273	B295731	1/50
Total-Seq-C0973	Biolegend	405275	B304487	1/50
Total-Seq-C0974	Biolegend	405277	B304481	1/50

Supplementary Table 6: Antibodies used in this study. pHLA: peptide HLA multimer assay, PC: phenotypic characterization by flow cytometry, PS: peptide stimulation assay, RNA-seq: sc-RNA- and TCR-seq assay, N/A: not applicable.

Antibody	Fluorochrome / DNA oligo	Company	Cat. No.	Clone	Lot. No.	Dilution	Assay
CCR7	BV711	BD	563712	150503	9022605	1/133	PC
CD14	APC-H7	BD	560180	MφP9	85600	1/100	pHLA, PS, PC, RNA-seq
CD14	FITC	BD	345784	MφP9	156613	1/100	pHLA, RNA-seq
CD16	APC-H7	BD	560715	3G8	9309425	1/100	pHLA, PS, PC, RNA-seq
CD16	BUV496	BD	612944	3G8	9213597	1/100	pHLA
CD16	FITC	BD	335035	NKP15	7003932	1/100	RNA-seq
CD19	APC-H7	BD	560177	SJ25C1	140610	1/100	pHLA, PS, PC, RNA-seq
CD19	BUV661	BD	750536	SJ25C1	288444	1/100	pHLA
CD27	BV421	BD	562514	M-T271	5051571	1/100	PC
CD28 and β2m	Total-Seq-C0251	BioLegend	394661	N/A	B282243	1/100	RNA-seq
CD28 and β2m	Total-Seq-C0253	BioLegend	394665	N/A	B282244	1/100	RNA-seq
CD28 and β2m	Total-Seq-C0254	BioLegend	394667	N/A	B282246	1/100	RNA-seq
CD28 and β2m	Total-Seq-C0255	BioLegend	394669	N/A	B282245	1/100	RNA-seq
CD28 and β2m	Total-Seq-C0257	BioLegend	394673	N/A	B306454	1/100	RNA-seq
CD4	APC-H7	BD	641398	SK3	35560	1/100	pHLA, PS, PC, RNA-seq
CD4	BB700	BD	566393	SK3	8248607	1/100	pHLA
CD4	FITC	BD	345768	SK3	6313547	1/100	RNA-seq
CD45RA	BUV563	BD	612926	HI100	7219651	1/400	PC
CD69	BUV395	BD	564364	FN50	7108931	1/50	PC
CD73	BB700	BD	746000	AD2	8229890	1/100	PC
CD8	BUV805	BD	612889	SK1	86704	1/50	pHLA, PS, PC
CD8	BV421	BD	562428	RPA-T8	9254861	1/50	RNA-seq
CD95	BUV737	BD	612790	DX2	7032582	1/50	PC
CXCR3	APC	BioLegend	353708	G025H7	7121923	1/100	PS
CXCR5	BV786	BioLegend	356936	J252D4	B245355	1/200	PC
HLA-DR	BUV661	BD	612980	G46-6	7249926	1/400	PC
IFNg	APC	BD	554702	B27	31187	1/50	PS
IL-17	PE	Biolegend	512306	BL168	B206920	1/100	PS
IL-2	BV750	BD	566361	MQ1-17H12	64351	1/200	PS
LAG-3	BV605	BD	745160	T47-530	8233745	1/100	PC
NKG2A	PE-Cy7	Beckman	B10246	Z199	200051	1/100	pHLA
PD-1	BUV737	BD	612791	EH12.1	9212298	1/100	pHLA
PD-1	PE-Cy7	BD	561272	EH12.1	0022949	1/100	PC
pHLA	APC	N/A	N/A	N/A	N/A	1/50	pHLA
pHLA	APC-R700	N/A	N/A	N/A	N/A	1/100	pHLA
pHLA	BB630	N/A	N/A	N/A	N/A	1/100	pHLA
pHLA	BB790	N/A	N/A	N/A	N/A	1/100	pHLA
pHLA	BUV395	N/A	N/A	N/A	N/A	1/100	pHLA
pHLA	BUV563	N/A	N/A	N/A	N/A	1/100	pHLA
pHLA	BUV615	N/A	N/A	N/A	N/A	1/100	pHLA, PC
pHLA	BV421	N/A	N/A	N/A	N/A	1/100	pHLA
pHLA	BV480	N/A	N/A	N/A	N/A	1/100	pHLA, PC
pHLA	BV605	N/A	N/A	N/A	N/A	1/100	pHLA
pHLA	BV650	N/A	N/A	N/A	N/A	1/100	pHLA
pHLA	BV711	N/A	N/A	N/A	N/A	1/100	pHLA
pHLA	BV750	N/A	N/A	N/A	N/A	1/100	pHLA
pHLA	PE	N/A	N/A	N/A	N/A	1/100	pHLA, PC
pHLA	Total-Seq-C0971	N/A	N/A	N/A	N/A	1/100	RNA-seq
pHLA	Total-Seq-C0972	N/A	N/A	N/A	N/A	1/100	RNA-seq
pHLA	Total-Seq-C0973	N/A	N/A	N/A	N/A	1/100	RNA-seq
pHLA	Total-Seq-C0974	N/A	N/A	N/A	N/A	1/100	RNA-seq
TIGIT	PerCP-eF710	eBioscience	46-9500-42	MBSA43	4318928	1/100	PC
TIM3	BV650	BD	565564	7D3	7241582	1/200	PC
TNF	FITC	BD	554512	MAb11	15360	1/50	PS

Supplementary References

1. Bakker, A. H. *et al.* Conditional MHC class I ligands and peptide exchange technology for the human MHC gene products HLA-A1, -A3, -A11, and -B7. *Proc. Natl. Acad. Sci. U.S.A.* **105,** 3825–3830 (2008).
2. Toebes, M. *et al.* Design and use of conditional MHC class I ligands. *Nat Med* **12,** 246–251 (2006).
3. Chang, C. X. L. *et al.* Conditional ligands for Asian HLA variants facilitate the definition of CD8+ T-cell responses in acute and chronic viral diseases. *Eur. J. Immunol.* **43,** 1109–1120 (2013).
