# Supplementary material for: Identification and characterization of a SARS-CoV-2 specific CD8+ T cell response with immunodominant features
Source: Nat Commun. 2021 May 10;12:2593. doi: 10.1038/s41467-021-22811-y (PMC8110804; doi:10.1038/s41467-021-22811-y)
Supplement: Supplementary file 3 — Descriptions of Additional Supplementary Files [file 41467_2021_22811_MOESM3_ESM.pdf]

Descriptions of Additional Supplementary Files

Supplementary data 1
Description: List of epitopes used for the identification of SARS-CoV-2-specific CD8 T cells.

Supplementary data 2
Description: Differential gene expression of Louvain clusters.

Supplementary data 3
Description: Gene signatures used to identify CD8 T cell differentiation states.

Supplementary data 4
Description: Differential gene expression and gene ontology of TTD-specific vs bulk naïve CD8 T cells.

Supplementary data 5
Description: Differential gene expression of TTD-specific vs bulk non-naïve CD8 T cells.
