# Supplementary material for: Identification and characterization of a SARS-CoV-2 specific CD8+ T cell response with immunodominant features
Source: Nat Commun. 2021 May 10;12:2593. doi: 10.1038/s41467-021-22811-y (PMC8110804; doi:10.1038/s41467-021-22811-y)
Supplement: Supplementary file 9 — Reporting Summary [file 41467_2021_22811_MOESM9_ESM.pdf]

nature research

Corresponding author(s): Pia Kvistborg

Last updated by author(s): Mar 19, 2021

Reporting Summary

Nature Research wishes to improve the reproducibility of the work that we publish. This form provides structure for consistency and transparency in reporting. For further information on Nature Research policies, see our Editorial Policies and the Editorial Policy Checklist.

Statistics

For all statistical analyses, confirm that the following items are present in the figure legend, table legend, main text, or Methods section.

n/a	Confirmed	
☐	☒	The exact sample size (n) for each experimental group/condition, given as a discrete number and unit of measurement
☐	☒	A statement on whether measurements were taken from distinct samples or whether the same sample was measured repeatedly
☐	☒	The statistical test(s) used AND whether they are one- or two-sided<br>*Only common tests should be described solely by name; describe more complex techniques in the Methods section.*
☐	☒	A description of all covariates tested
☐	☒	A description of any assumptions or corrections, such as tests of normality and adjustment for multiple comparisons
☐	☒	A full description of the statistical parameters including central tendency (e.g. means) or other basic estimates (e.g. regression coefficient) AND variation (e.g. standard deviation) or associated estimates of uncertainty (e.g. confidence intervals)
☐	☒	For null hypothesis testing, the test statistic (e.g. F, t, r) with confidence intervals, effect sizes, degrees of freedom and P value noted<br>*Give P values as exact values whenever suitable.*
☒	☐	For Bayesian analysis, information on the choice of priors and Markov chain Monte Carlo settings
☒	☐	For hierarchical and complex designs, identification of the appropriate level for tests and full reporting of outcomes
☐	☒	Estimates of effect sizes (e.g. Cohen's d, Pearson's r), indicating how they were calculated

Our web collection on statistics for biologists contains articles on many of the points above.

Software and code

Policy information about availability of computer code

Data collection	BD FACSDiva v.8.0.1, acquisition and collection of Flow Cytometry data<br>R version v.3.6.3, SARS-CoV-2 epitope prediction<br>data.table v.1.12.6, SARS-CoV-2 epitope prediction<br>NetMHCpan-4.0, predict binding affinity of SARS-CoV-2 epitopes to the MHC<br>NetChop-3.1, predict proteasomal processing of SARS-CoV-2 epitopes

Data analysis	BD FACSDiva v.8.0.1, analysis of Flow Cytometry data<br>FlowJo v.10.6.2, analysis of Flow Cytometry data<br>Excel v.16.36, Statistical analysis<br>PRISM v.8.4.0, Statistical analysis<br>Cell Ranger Software Suite v.3.1.0, scRNAseq data de-multiplexing, barcode processing, single-cell 5' UMI counting and TCR sequence assembly<br>Scanpy v.1.5.1, sc-RNAseq data analysis<br>Python v.3.7.6, sc-RNAseq data manipulation<br>Pandas v.1.0.1, sc-RNAseq data manipulation<br>NumPy v.1.18.1, sc-RNAseq data manipulation<br>Seaborn v.0.10.0, sc-RNAseq data plotting<br>Matplotlib v.3.1.3, sc-RNAseq data plotting<br>DAVID Bioinformatics Resources v.6.8, gene ontology analysis<br>Cell Ranger vdj pipeline v.3.1.0, TCR sequence assembly<br>Scirpy v.0.3, sc-TCR-seq data analysis<br>COVID-19 CoV Genomics tool v.1.6.0, identification of SNVs across the SARS-CoV-2 genome

For manuscripts utilizing custom algorithms or software that are central to the research but not yet described in published literature, software must be made available to editors and reviewers. We strongly encourage code deposition in a community repository (e.g. GitHub). See the Nature Research guidelines for submitting code & software for further information.

Data

Policy information about availability of data

All manuscripts must include a data availability statement. This statement should provide the following information, where applicable:

- Accession codes, unique identifiers, or web links for publicly available datasets
- A list of figures that have associated raw data
- A description of any restrictions on data availability

Flow cytometry data generated in this study are deposited on FlowRepository with the identifier FR-FCM-Z3KA. RNA-sequencing data generated in this study are deposited in the Gene Expression Omnibus (GEO) repository with the accession code (available latest 26-Mar-2021). Source data for Fig. 1d and e, Fig. 2a and c, Fig. 3d and e, Fig, 5b, d and f are provided with the paper.

Field-specific reporting

Please select the one below that is the best fit for your research. If you are not sure, read the appropriate sections before making your selection.

☒ Life sciences ☐ Behavioural & social sciences ☐ Ecological, evolutionary & environmental sciences

For a reference copy of the document with all sections, see nature.com/documents/nr-reporting-summary-flat.pdf

Life sciences study design

All studies must disclose on these points even when the disclosure is negative.

Sample size	No sample size calculation was performed, all samples available for analysis were included.<br><br>Peripheral blood mononuclear cell (PBMC) samples from 39 COVID-19 patients with critical (n=18), severe (n=12), moderate (n=2), and asymptomatic disease (n=7) were used. All COVID-19 patients were tested positive for SARS-CoV-2 using reverse transcriptase polymerase chain reaction (RT-PCR) from an upper respiratory tract (nose/throat) swab test in accredited laboratories. In addition, PBMC samples from 7 healthy individuals, collected prior to November 2019, were used.
Data exclusions	The following exclusion criteria were established prior to conducting the pHLA multimer assay: no HLA coverage (n=4) or failed HLA typing (n=1).<br><br>The following exclusion criteria were established prior the analysis of antigen-specific CD8 T cell responses: insufficient CD8+ T cell counts (<1000 cells, n=3).
Replication	SARS-CoV-2 CD8 T cell reactivity: if sufficient material was available, a response was confirmed using a different streptavidin-conjugated fluorochrome combination for the same peptide-HLA, to make sure the response is not a result of fluorescence artifacts. This confirmation could be done and was successful for patients COVID-004, 087, 096, 121, 127, 143 and 153. To check if a response was not as a result of unspecific staining, PBMCs from two COVID-19 patients (n=4) that we could not screen due to non-matching HLA types were screened with peptide-HLA combinations that gave a response.<br><br>SARS-CoV-2 CD8 T cell functionality: intracellular cytokine staining was performed upon specific peptide stimulation. The experiment was performed twice for 2 patients (COVID-143 and COVID-153) with similar results.
Randomization	There was no randomization done in this work.
Blinding	Only experimental blinding is applicable for this study: blinding regarding patient characteristics (age, sex, disease stage, HLA type, design of

dual fluorescent codes) during the analysis of SARS-CoV-2-specific CD8 T cell responses.

Reporting for specific materials, systems and methods

We require information from authors about some types of materials, experimental systems and methods used in many studies. Here, indicate whether each material, system or method listed is relevant to your study. If you are not sure if a list item applies to your research, read the appropriate section before selecting a response.

Materials & experimental systems

n/a	Involved in the study
☐	☒ Antibodies
☒	☐ Eukaryotic cell lines
☒	☐ Palaeontology and archaeology
☒	☐ Animals and other organisms
☐	☒ Human research participants
☒	☐ Clinical data
☒	☐ Dual use research of concern

Methods

n/a	Involved in the study
☒	☐ ChIP-seq
☐	☒ Flow cytometry
☒	☐ MRI-based neuroimaging

Antibodies

Antibodies used

Streptavidin-conjugated reagents:
SA-APC (Invitrogen, cat: S868, lot: 1876191), SA-APC-R700 (BD, cat: 565144, lot: 9023546), SA-BB630 (BD, cat: custom, lot: 0091407), SA-BB790 (BD, cat: custom, lot: 0091415), SA-BUV395 (BD, cat: 564176, lot: 9078721), SA-BUV563 (BD, cat: 567655, lot: 9227337), SA-BUV615 (BD, cat: 613013, lot: 9193969), SA-BV421 (BD, cat: 563259, lot: 9197684), SA-BV480 (BD, cat: 564876, lot: 9115527), SA-BV605 (BD, cat: 563260, lot: 9119807), SA-BV650 (BD, cat: 563855, lot: 9197600), SA-BV711 (BD, cat: 563262, lot: 0007729), SA-BV750 (BD, cat: custom, lot: 0091421), SA-PE (Invitrogen, cat: S866, lot: 1736956), Total-Seq-C091 (BioLegend, cat: 405271, lot: B295732), Total-Seq-C092 (BioLegend, cat: 405273, lot: B295731), Total-Seq-C093 (BioLegend, cat: 405275, lot: B304487), Total-Seq-C093 (BioLegend, cat: 405274, lot: B304481).

Antibodies:
aCCR7-BV711 (BD, cat: 563712, clone: 150503, lot: 9022605), aCD14-APC-H7 (BD, cat: 560180, clone: MϕP9, lot: 85600), aCD14-FITC (BD, cat: 345784, clone: MϕP9, lot: 156613), aCD16-APC-H7 (BD, cat: 560715, clone: 3G8, lot: 9309425), aCD16-BUV496 (BD, cat: 612944, clone: 3G8, lot: 9213597), aCD16-FITC (BD, cat: 335035, clone: NKP15, lot: 7003932), aCD19-APC-H7 (BD, cat: 560177, clone: SJ25C1, lot: 140610), aCD19-BUV661 (BD, cat: 750536, clone: SJ25C1, lot: 288444), aCD19-FITC (BD, cat: 345776, clone: 4G7, lot: 6328889), aCD27-BV421 (BD, cat: 562514, clone: M-T271, lot: 5051571), aCD4-APC-H7 (BD, cat: 641398, clone: SK3, lot: 35560), aCD4-BB700 (BD, cat: 566393, clone: SK3, lot: 8248607), aCD4-BUV496 (BD, cat: 612937, clone: SK3, lot: 8277620), aCD4-FITC (BD, cat: 345768, clone: SK3, lot: 6313547), aCD45RA-BUV563 (BD, cat: 612926, clone: HI100, lot: 7219651), aCD69-BUV395 (BD, cat: 564364, clone: FN50, lot: 7108931), aCD73-BB700 (BD, cat: 746000, clone: AD2, lot: 8229890), aCD8-BUV805 (BD, cat: 612889, clone: SK1, lot: 86704), aCD8-BV421 (BD, cat: 562428, clone: RPA-T8, lot: 9254861), aCD95-BUV737 (BD, cat: 612790, clone: DX2, lot: 7032582), aCXCR3-APC (BioLegend, cat: 353708, clone: G025H7, lot: 7121923), aCXCR5-BV785 (BioLegend, cat: 356936, clone: J252D4, lot: B245355), aHLA-DR-BUV661 (BD, cat: 612980, clone: G46-6, lot: 7249926), aIFNg-APC (BD, cat: 554702, clone: B27, lot: 31187), aIL-17-PE (Biolegend, cat: 512306, clone: BL168, lot: B206920), aIL-2-BV750 (BD, cat: 566361, clone: MQ1-17H12, lot: 64351), LIVE/DEAD Fixable IR Dead Cell Stain Kit (Invitrogen, cat: L10119, clone: N/A, lot: N/A), aLAG-3-BV605 (BD, cat: 745160, clone: T47-530, lot: 8233745), aNKG2A-PE-Cy7 (Beckman, cat: B10246, clone: Z199, lot: 200051), aPD-1-BUV737 (BD, cat: 612791, clone: EH12.1, lot: 9212298), aPD-1-PE-Cy7 (BD, cat: 561272, clone: EH12.1, lot: 0022949), aTIGIT-PerCP-eF710 (eBioscience, cat: 46-9500-42, clone: MBSA43, lot: 4318928), aTim-3-BV786 (BD, cat: 742857, clone: 7D3, lot: 112835), aTIM3-BV650 (BD, cat: 565564, clone: 7D3, lot: 7241582), aTNFa-FITC (BD, cat: 554512, clone: MAb11, lot: 15360), Total-Seq-C0251 (BioLegend, 394661, B282243), Total-Seq-C0253 (BioLegend, 394665, B282244), Total-Seq-C0254 (BioLegend, 394667, B282246), Total-Seq-C0255 (BioLegend, 394669, B282245), Total-Seq-C0257 (BioLegend, 394673, B306454).

Validation

BD: The antibody development process includes testing on a combination of primary cells, cell lines and/or transfectant cell models with relevant controls using multiple immunoassays to ensure biological accuracy. They perform multiplexing with additional antibodies to interrogate antibody staining in multiple cell populations.

BioLegend: All newly developed clones at BioLegend undergo validation testing for multiple applications. This serves as a cross-check for specificity and provides clarity for research uses. Typically, antibodies are tested by two or more of the following methods: endogenous positive and negative expressing cells, KO/KD cell lines, isotype controls, biological testing (ie. treatments, activation etc.), multiple donor samples for human cells, side-by-side comparisons with currently available clones. This is followed by Western-Blotting, Flow Cytometry, ChIP, Immunofluorescence imaging, Immunohistochemistry.

Invitrogen: Antibodies are being tested using at least 1 of the following methods to ensure proper functionality in researchers' experiments: knockout, knockdown, independent antibody verification (IAV), cell treatment, relative expression, neutralization, peptide array, SNAP-ChIP, Immunoprecipitation-Mass spectrometry (IP-MS). This is followed by Western-Blotting, Flow Cytometry, ChIP, Immunofluorescence imaging, Immunohistochemistry.

Beckman Coulter: Beckman Coulter offers the largest portfolio of CE-IVD and ASR conjugated antibodies validated against clinical standards. We develop and manufacture reagents according to current Good Manufacturing Practices (cGMP), the highest quality standards in the industry, ensuring optimal antibody panel performance.

Human research participants

Policy information about studies involving human research participants

Population characteristics	All available samples from COVID-19 patients (positive SARS-CoV-2- PCR test) without any inclusion/exclusion criteria have been collected.<br><br>Samples from healthy donors were collected before November 2019. Inclusion criteria for healthy donors were: no evidence for ongoing disease, no immunosuppressive medication.
Recruitment	Samples were collected within a local biobanking effort of COVID-19 samples.
Ethics oversight	Samples were collected in accordance with the Declaration of Helsinki after approval by the institutional review boards (Ethical Committee of Area Vasta Emilia Romagna, protocol number 177/2020, March 10th, 2020, and subsequent amendments).

Note that full information on the approval of the study protocol must also be provided in the manuscript.

Flow Cytometry

Plots

Confirm that:

- [x] The axis labels state the marker and fluorochrome used (e.g. CD4-FITC).
- [x] The axis scales are clearly visible. Include numbers along axes only for bottom left plot of group (a 'group' is an analysis of identical markers).
- [x] All plots are contour plots with outliers or pseudocolor plots.
- [x] A numerical value for number of cells or percentage (with statistics) is provided.

Methodology

Sample preparation

PBMC islation and storage:
Peripheral blood was collected in ethylenediaminetetraacetic acid (EDTA) tubes following subsequent isolation of PBMCs using Ficoll-Paque density centrifugation according to standard protocol. PBMCs were suspended in fetal bovine serum (FCS, Sigma, F7524) with 1dimethyl sulfoxide (DMSO, Sigma, D4540, 10% v/v) and stored in liquid nitrogen.

pHLA multimer assay and phenotypic characterization:
For the pHLA multimer assay and the phenotypic characterization, PBMC samples were thawed and washed with RPMI 1640 (Life Technologies, 21875-034) supplemented with human serum (Sigma, H3667, 10% v/v), penicillin-streptomycin (Life Technologies, 15140-122, 1% v/v) and benzonase nuclease (Merck-Millipore, 70746-4, 2500 U/mL), resuspended and incubated at 37˚C for 30 minutes before staining. Antigen-specific CD8 T cells were stained for 15 min at 37 °C with pHLA multimers (Supplementary Table 6) encoding unique dual fluorescent code combinations for up to 75 epitopes. Subsequently, cells were stained for 20 min on ice with antibodies (Supplementary Table 6). LIVE/DEAD Fixable IR Dead Cell Stain Kit (Invitrogen, L10199) staining was performed for 20 min on ice either during antibody staining (pHLA multimer assay, 1/200) or for 10 min on ice after antibody staining (phenotypic characterization, 1/400). Individual staining was performed in the presence of Brilliant Staining Buffer Plus (BD, 563794) according to manufacturer's protocol (BD) and samples were washed twice before acquisition.

Peptide stimulation assay:
PPBMCs were thawed, washed and incubated at 37˚C for 30 minutes or at 4˚C for 60 minutes (if cells were simultaneously used for single-cell RNA sequencing) in RPMI 1640 (Life Technologies, 21875-034) supplemented with human serum (Sigma, H3667, 10% v/v), penicillin-streptomycin (Life Technologies, 15140-122, 1% v/v) and benzonase nuclease (Merck-Millipore, 2500 U/mL). After washing, equal amounts of PBMCs (≥ 1x105 cells per condition) were cultured for 12 hours at 37˚C in the presence of GolgiPlug (BD, 555029, 1/1000), and either the TTDPDFLGRY peptide (2 µg/mL) or equimolar amounts of DMSO (negative control). Phorbol 12-myristate 13-acetate (50 ng/mL) and Ionomycin (1 µg/mL) were used as technical control. Cells were washed and stained for 20 min on ice with surface marker antibodies (Supplementary Table 6). After washing, cells were stained for 10 min on ice with LIVE/DEAD Fixable IR Dead Cell Stain Kit (Invitrogen, L10119, 1/400). Subsequently, cells were washed, fixed and permeabilized using the Foxp3 Transcription Factor Staining Buffer Set (eBioscience, 00-5523-00) according to manufacturer's protocol. Intracellular cytokines were stained for 20 min on ice with antibodies (Supplementary Table 6). Cells were washed twice before acquisition.

Sorting of SARS-CoV-2 specific CD8 T cells:
PBMCs were thawed, washed and incubated for 60 min on ice in cold RPMI 1640 (Life Technologies, 21875-034) supplemented with human serum (Sigma, H3667, 10% v/v), penicillin-streptomycin (Life Technologies, 15140-122, 1% v/v) and benzonase nuclease (Merck-Millipore, 70746-4, 2500 U/mL). TotalSeqTM-streptavidin oligo barcoded reagents (Supplementary Table 5) were conjugated to pHLA monomers (100g/ml) as described above and used to stain SARS-CoV-2-specific CD8 T cells for 30 min on ice (Supplementary Table 6). Subsequently cells were stained for 20 min on ice with antibodies (Supplementary Table 6) and LIVE/DEAD Fixable IR Dead Cell Stain Kit (Invitrogen, L10119, 1/200). Stained cells from individual patients were pooled and washed before sorting on the FACSAria Fusion.

Instrument	BD FACSymphony A5<br>FACSAria Fusion
Software	BD FACSDiva v.8.0.1, acquisition and collection of Flow Cytometry data<br>FlowJo 10.6.2, analysis of Flow Cytometry data
Cell population abundance	Two post-sort sample: 22,364 (batch I) and 11,747 cells (batch II) D8 T cells according to FACSDiva.
Gating strategy	Combinatorial encoding of pHLA multimers and surface marker staining<br>The following gating strategy was applied to identify CD8+ T cells: (i) selection of live (IRDye low-dim) single-cell lymphocytes [forward scatter (FSC)-W/H low, side scatter (SSC)-W/H low, FSC/SSC-A], (ii) selection of anti-CD8+ and 'dump' (anti-CD4, anti-CD14, anti-CD16, anti-CD19) negative cells. Antigen-specific CD8 T cell responses that were positive for only 2 and none of the other pHLA multimer channels were identified using Boolean gating. All gates for phenotypic characterization of bulk CD8 T cells and SARS-CoV-2-specific CD8 T cells were set based on bulk CD8 T cells.<br><br>Peptide stimulation assay<br>The following gating strategy was applied to identify CD8+ T cells: (i) selection of live (IRDye low-dim) single-cell lymphocytes [forward scatter (FSC)-W/H low, side scatter (SSC)-W/H low, FSC/SSC-A], (ii) selection of anti-CD8+ and 'dump' (anti-CD4, anti-CD14, anti-CD16, anti-CD19) negative cells.<br><br>Sorting of (SARS-CoV-2-specific) CD8 T cells<br>The following gating strategy was applied to identify and sort CD8+ T cells into PBS supplemented with 0.04% bovine serum albumin (w/v) at 4˚C: (i) selection of live (IRDye low-dim) single-cell lymphocytes [forward scatter (FSC)-W/H low, side scatter (SSC)-W/H low, FSC/SSC-A], (ii) selection of anti-CD8+ positive and 'dump' (anti-CD4, anti-CD14, anti-CD16, anti-CD19) negative cells.

☒ Tick this box to confirm that a figure exemplifying the gating strategy is provided in the Supplementary Information.
